# Value network analysis for facilitator development in project-based learning

**DOI:** 10.1016/j.mex.2024.102846

**Published:** 2024-07-09

**Authors:** Iniga Antonia Baum, Christian Stary

**Affiliations:** Johannes Kepler University, Business Informatics-Communications Engineering, Business School, Linz, Austria

**Keywords:** Value network analysis, Project-based learning, Facilitator development, Organizational learning, Value Network Analysis for Project-based Learning (VNA/P)

## Abstract

The method enhances Value Network Analysis (VNA) in the context of Project-Based Learning (PjBL). Utilizing the appropriated VNA, facilitators can reflect and continuously improve their learning support in an institutional (learning) setting. Thereby,

• PjBL frames the VNA application through success factors and guidelines for effective PjBL practice
• PjBL success factors and guidelines are
  ∘ linked to value transactions among PjBL stakeholders
  ∘ considered from a facilitator's perspective
• PjBL is advanced in a transparent and participatory way.

In order to make existing potential for change tangible, the method leads to developing proposals as substantiated offers to other stakeholders. Once getting accepted on the organizational level, their implementation completes the intended collective learning step.

## Graphical abstract

**Figure:**
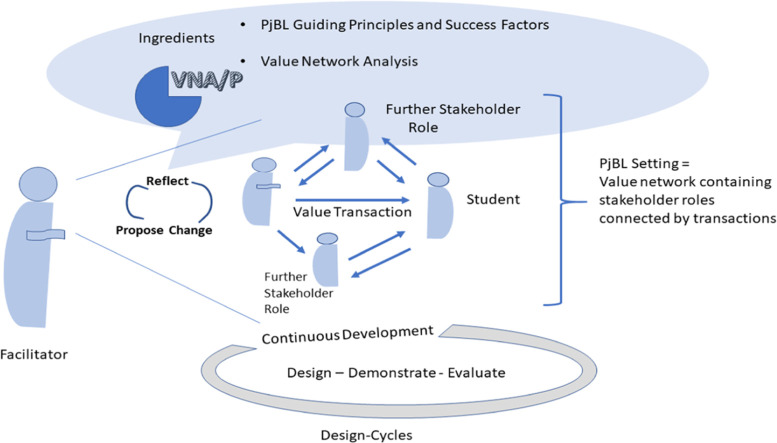

Specifications table

**Table:** 

Subject area:	Economics and Finance
More specific subject area:	Facilitator Development in Project-based Learning
Name of your method:	Value Network Analysis for Project-based Learning (VNA/P)
Name and reference of original method:	Allee, V. [2]. The Future of Knowledge: Increasing Prosperity through Value Networks. Amsterdam: Butterworth-Heinemann. Allee, V. [3]. Value Network Analysis and value conversion of tangible and intangible assets. Published in Journal of Intellectual Capital, Volume 9, No. 1, 2008, 5–24. Allee, V. [4]. Value‐creating networks: Organizational issues and challenges. The Learning Organization, 16(6), 2009, 427–442.
Resource availability:	Baum, I.A., Stary, C. Value-driven Facilitation of Project-based Learning: Domain Knowledge, Didactic Guidance, and Method Support, Proc. International Conference on Knowledge Management, Florianopolis, 2023, https://www.researchgate.net/profile/Christian-Stary/publication/375748156_Value-driven_Facilitation_of_Project-based_Learning_Domain_Knowledge_Didactic_Guidance_and_Method_Support/links/65e44f21c3b52a117006e31b/Value-driven-Facilitation-of-Project-based-Learning-Domain-Knowledge-Didactic-Guidance-and-Method-Support.pdf or https://shorturl.at/g4nzg - link to the paper in the final proceedings ISSN 2594-7958. https://prezi.com/view/FHbGvjL42L6pn3g2It3p/ - link to tool support.

## Method details

### Appropriating value network analysis for effective facilitation of PjBL

We first provide insight into the context of applying the Value Network Analysis (VNA) by introducing the rationale and fundamental principles of facilitating Project-based Learning (PjBL) before detailing the steps of VNA/P. The abbreviation of the method VNA/P indicates the customization of VNA by PjBL.

PjBL approaches learning processes in a highly individualized and focused form. With goal-setting that concerns challenges and problems students may face in real settings, learners are encouraged to self-organize problem solving processes and collaboration while developing knowledge and skills through project practice (cf. [9,10,15]). Since PjBL has been recognized as student-driven, but teacher-facilitated approach to learning [13], we need to understand why facilitators respond differently to the same challenges, and what can be done to better support and sustain this type of constructivist learning processes (cf. [12]).

What has become evident from various PjBL implementations, e.g., in engineering education [8], facilitators experience several challenges [11,14]. These have been reported on the individual level for teachers and students, as well as on the institutional level and the culture level, calling for optimization of curricula design and informed preparation to implement PjBL successfully [7]. Facilitator understanding of PjBL in terms of adjusting to the role of project-oriented learner- and management support is crucial for its adoption.

VNA/P makes a step to overcome this lack of congruence and to enable collective development, as we want to uncover the facilitators' individual perception of practice as part of their interaction with students and other stakeholders considered relevant or valuable in their educational context. VNA/P has its focus on the following items:

(i) Which value exchanges do facilitators perceive when performing PjBL activities?
(ii) How can they be supported to implement PjBL according to the state of art and envisioned value transactions^1^?

**Fig. 1:**
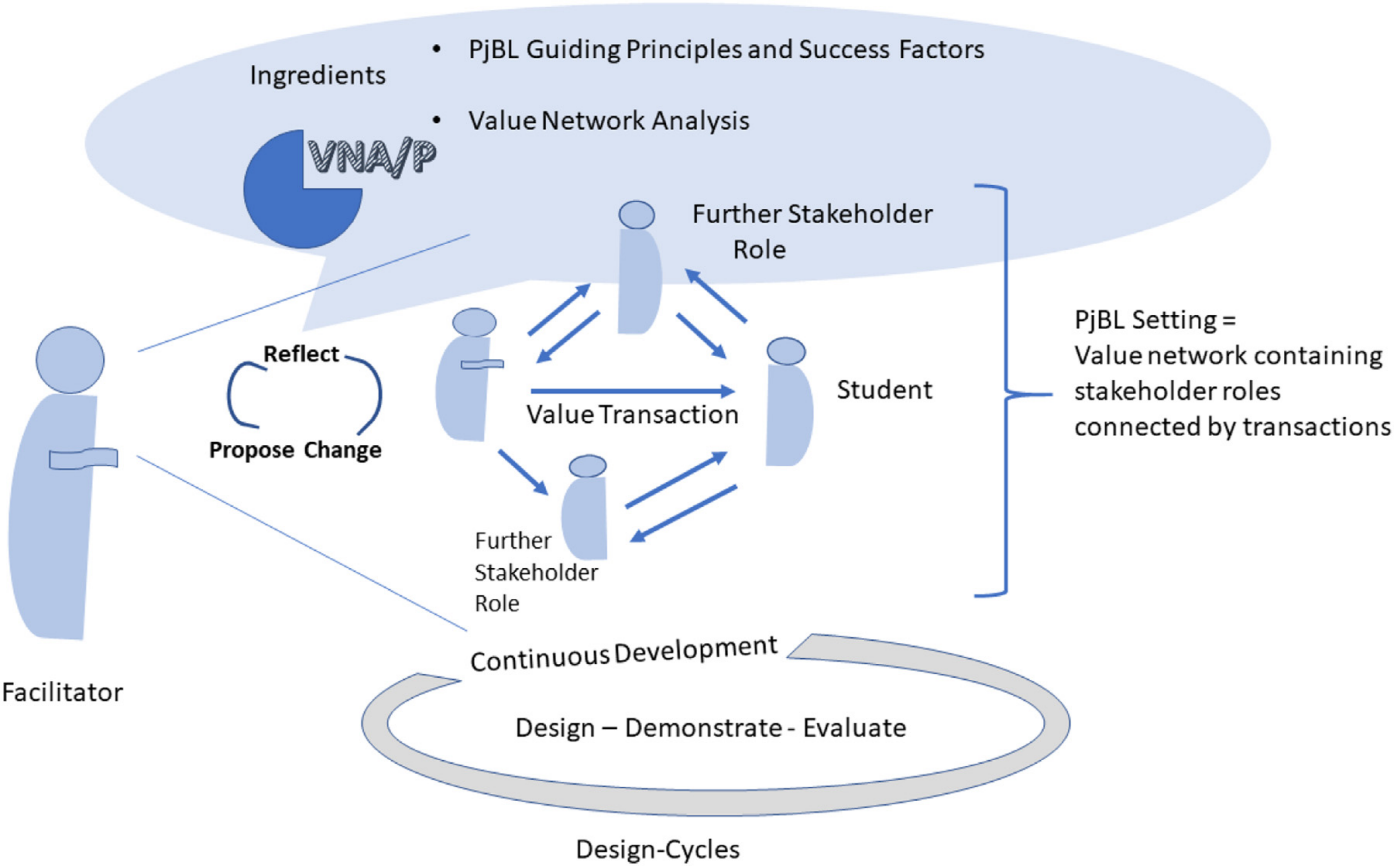
VNA/P operation based the exploration of value exchanges in PjBL.

In order to understand the value transactions that may support or hinder the connection with students and institutional actors, VNA/P addresses PjBL facilitator interactions along learning processes, in particular when performing PjBL activities in an educational setting. For eliciting, representing, and sharing of value transactions we utilize Value Network Analysis (VNA) [4], and frame it with experiential PjBL knowledge, as shown in Fig. 1.

The contextual frame is based on the following 8 success factors and guidelines as described in Baum et al. [6]. They have been identified through an extensive analysis of empirical findings on PjBL: Hence, PjBL should follow a set of essential principles **(PjBL-ESSENTIALS)** to succeed:

• An actively found and constructed problem by learners
• Situated learning in authentic context
• Collaborative learning
• Deployment of cognitive & technical tools
• Externally presented artifact
• Teacher as Facilitator ensuring learner-centricity of support
• Feedback to learners
• Reflection by all stakeholders

The Value Network Analysis targets a key question of (business) organizations, namely “How is value created through converting intangible assets to tangible ones, and thus negotiable forms of value?” [3]. The method aims at developing organizations beyond the value chain, since traditional value-chain models represent a linear, if not mechanistic, view of an organization and its operation.

Complex constellations of values, however, require analyzing stakeholder relationships by taking into account the role of knowledge and intangible value exchange as a foundation for value creation. Value exchange needs to be analyzed before changing (business) transactions in practice. In particular, complex relationships require pre-processing from a value-based perspective, as they influence effectiveness and efficiency, and cause possible friction in operational processes [3].

VNA is meant to be a development instrument beyond engineering, as it aims to understand organizational dynamics, and thus to manage structural knowledge from a value-seeking perspective, for individuals and the organization as a whole. However, it is based on several fundamental principles and assumptions [[1], [2], [3]]:

• Participants of an organization and organizationally relevant stakeholders participate in a value network by converting what they know, both individually and collectively, into tangible and intangible values that they contribute to the network, and thus to the organization.
• Participants accrue value from their participation by converting value inputs into positive increases of their tangible and intangible assets, in ways that will allow them to continue producing value outputs in the future.
• In such a network, each participant contributes and receives value in ways that sustain both their own success and the success of the value network as a whole. This mutual dependency is a *conditio sine qua non*. Once active participants either withdraw or are expelled, the overall system becomes unstable and may collapse, and needs to be reconfigured.
• Value networks require trusting relationships and a high level of integrity and transparency on the part of all participants. Then, insights can be gained into interactions by identifying and analyzing not only the patterns of exchange, but rather the impact of value transactions, exchanges, and flows, and thus the dynamics of creating and leveraging value.
• A single transaction is only meaningful in relation to the system as a whole. It is set by role carriers who utilize incoming deliverables from other role carriers (inputs) and can assess their value, and they realize value that is manifest by generating output.

As stakeholders – in relevant technical roles – are responsible for their relations with others, the organization itself is conceptualized as a highly dynamic and complex setting.

### VNA/P procedure

For each of the success factors and guiding principles the following VNA/P cycle (Step 2–6) should be performed:

**STEP 1: INFORM ON ESSENTIAL PjBL-PRINCIPLES (see PjBL-ESSENTIALS)**

According to the PjBL appropriation of the VNA, each user is asked to become aware and informed about the PjBL-ESSENTIALS as listed above under this label.

***FOR EACH OF THE PjBL-ESSENTIALS PERFORM STEP 2–6***

***STEP 2: DRAW OR ADAPT A HOLOMAP***

After having selected a PjBL essential, a diagrammatic representation of a PjBL setting is created in form of a value network. The diagrammatic representation is termed holomap. It is a network of PjBL actors and their relations. PjBL-relevant actors when implementing each of the PjBL essentials or reflecting on each of them, become nodes of the network, and actor's transactions become labelled directed edges connecting the nodes. The connections between actors can either be tangible (i.e. from the technical stakeholder role required), or intangible (i.e. not formally required but provided for the sake of successful PjBL processes). Thus, the connections are drawn either as tangible or intangible.

***STEP 3: PERFORM EXCHANGE ANALYSIS***

The created holomap shows patterns that are of interest in the first analysis of the VNA. The interaction patterns should reveal cyclic interactions between actors denoting value exchanges. This means, each delivered output should either lead to a direct or indirect input to the sending stakeholders. Such a pattern establishes a value exchange.

***STEP 4: PERFORM IMPACT ANALYSIS***

The second analysis of the VNA has its focus on transactions from each stakeholder pointing to the facilitator. The relevant details for the facilitator contributing the individual case are given in Table 1.

**Table 1:** Impact Analysis (adopted from [2]).

Transactions (Facilitator)			Impact Analysis							
Deliverable	From	To	Activities that are generated	Impact on financial resources +/-		Impact on intangible assets + / -		Overall cost/risk for this input	Overall benefit of this input	Perceived value in view of recipient
					*Human Competence*	*Internal* *Structure*	*(Business)* *Relationships*	*H = High* *M=Medium* *L = Low*	*H = High* *M=Medium* *L = Low*	*+2, +1* *Neutral* *−1, −2*

***STEP 5: PERFORM VALUE CREATION ANALYSIS***

The third analysis of the VNA has its initial focus on outgoing deliverables from the facilitator to different stakeholders of the value network and its second focus on additional value transactions to improve the collective performance of the network, even by complementing additional stakeholders. Its details for the facilitator contributing data from the individual case including offers to other stakeholders are given in Table 2.

**Table 2:** Value Creation Analysis (adopted from [2]).

**Transactions** (Facilitator)			**Perceived Value**					
**Deliverable**	**From**	**To**	**Recipient highly values this deliverable.** **Strongly agree (+2)** **Agree (+1)** **Neutral (0)** **Disagree (−1)** **Strongly disagree (−2)**	**Value Creation Analysis**				
				**Tangible asset utilization is:** ***H* = High.** ***M* =Medium.** ***L* = Low**	**What are the tangible costs? (concerning financial and physical resources)**	**How high is the risk factor in providing this output?.** ***H* = High.** ***M* = Medium.** ***L* = Low**	**How high is the risk factor in providing this output?.** ***H* = High.** ***M* = Medium.** ***L* = Low**	

**Value Creation Analysis (continued)**								

**Intangible asset utilization is:** ***H* = High, *M* = Medium *L* = Low** **HC = Human Competence** **IS = Internal Structures** **BR = (Business) Relationships**			**What are other intangible costs or benefits? (Industry, society, environment)**			**How do we add to, enhance, or extend value?**	**What is the overall combined cost/risk for this input?**	**What is the overall benefit for us in providing this input?**
*Human Competence*	*Internal Structure*	*(Business) Relationships*	*Organizations*	*Society*	*Environment*	*Who else could profit?*		

***STEP 6: ADAPT HOLOMAP UPON STAKEHOLDER ACCEPTANCE***

The final step reconsiders the initial holomap adapting it to each of the offers made to other stakeholders, as documented in the Value Creation Analysis when being asked **How do we add to, enhance, or extend value?** (see Table 2), and once being accepted by addressed stakeholders. The provided offers can be new deliverables, additional receivers, or transactions.

The contextual framing of the VNA through PjBL essentials along STEP 1–6 has been tested in the field with educators from various academic institutions, from the perspective of PjBL in classroom teaching, curriculum design, and institutional development – see also Baum et al. [5,6]. The moderation of the use cases has been performed by the authors, receiving feedback for both, the methodological approach, and user experience.

## Ethics statements

We confirm that the relevant informed consent was obtained from all subjects involved in validating VNA/P.

## CRediT authorship contribution statement

**Iniga Antonia Baum:** Conceptualization, Methodology, Software, Data curation. **Christian Stary:** Visualization, Validation, Writing – review & editing.

## Declaration of Competing Interest

The authors declare that they have no known competing financial interests or personal relationships that could have appeared to influence the work reported in this paper.

## Data Availability

Data will be made available on request.

## Appendix Supplementary materials

Image, application 1

## Footnotes

1 A value transaction is the exchange of information, material, or services between stakeholders in a specific role (student, facilitator, coach, expert, educators, and the like) considered to be relevant for PjBL or contributing to successful PjBL.
