# Supplementary material for: Value network analysis for facilitator development in project-based learning
Source: MethodsX. 2024 Jul 9;13:102846. doi: 10.1016/j.mex.2024.102846 (PMC11298650; doi:10.1016/j.mex.2024.102846)
Supplement: Supplementary file 1 [file mmc1.docx]

MathodX-Supplementary Material

**Supplementary material *and* additional information**

- Project-based Learning works - <https://www.pblworks.org/what-is-pbl>
- Guide to Project-based Learning - <https://www.teacheracademy.eu/blog/project-based-learning/>
- Verna Allee describes Value Networks - <https://www.youtube.com/watch?v=VC7W8cMiVFo>
- Verna Allee on making implicit knowledge explicit – <https://www.youtube.com/watch?v=lPNC0aLIXzI>
- Networks Inspiring Interviews - <https://www.youtube.com/watch?v=vk36KSlr7oc>
- Alexander Schieffer (2004) Value networks: how organisations really work An Interview with Verna Allee, Knowledge Management Research & Practice, 2:3, 194-199, DOI: [10.1057/palgrave.kmrp.8500041](https://doi.org/10.1057/palgrave.kmrp.8500041)
- Christian Stary (2014) Non-disruptive knowledge and business processing in knowledge life cycles – aligning value network analysis to process management, [*Journal of Knowledge Management*](https://www.emerald.com/insight/publication/issn/1367-3270), Vol. 18 No. 4, pp. 651-686. <https://doi.org/10.1108/JKM-10-2013-0377>
